# Efficacy and safety of atezolizumab–bevacizumab vs pembrolizumab-lenvatinib in unresectable hepatocellular carcinoma: a retrospective, cohort study

**DOI:** 10.3389/fimmu.2024.1472870

**Published:** 2024-11-19

**Authors:** Zili Hu, Zhoutian Yang, Zibo Fu, Yizhen Fu, Zeliang Ma, Zhongguo Zhou, Minshan Chen, Yaojun Zhang

**Affiliations:** ^1^ Department of Liver Surgery, Sun Yat-Sen University Cancer Center, Guangzhou, China; ^2^ Collaborative Innovation Center for Cancer Medicine, State Key Laboratory of Oncology in South China, Sun Yat-Sen University Cancer Center, Guangzhou, China; ^3^ Guangdong Provincial Clinical Research Center for Cancer, Sun Yat-Sen University Cancer Center, Guangzhou, China; ^4^ Department of Stomatology, The First Affiliated Hospital of Sun Yat-Sen University, Guangzhou, China; ^5^ Department of Oncology, National Cancer Center/National Clinical Research Center for Cancer/Cancer Hospital, Chinese Academy of Medical Sciences and Peking Union Medical College, Beijing, China

**Keywords:** unresectable hepatocellular carcinoma, atezolizumab, bevacizumab, pembrolizumab, lenvatinib

## Abstract

**Background:**

The relative superiority of atezolizumab–bevacizumab versus pembrolizumab-lenvatinib in treatment of unresectable hepatocellular carcinoma (HCC) remains uncertain. This study aims to compare the efficacy of atezolizumab–bevacizumab and pembrolizumab-lenvatinib in first-line treatments for unresectable HCC.

**Methods:**

A total of 72 patients receiving pembrolizumab-lenvatinib (PL group) and 92 patients receiving atezolizumab-bevacizumab (AB group) between January 2019 and June 2023 were included in this study. By employing propensity score matching (PSM), we compared the overall survival (OS) and progression-free survival (PFS) between the two groups.

**Results:**

After PSM, the 1-, 2-, and 3-year OS rates were 70.4%, 54.5%, and 40.0% in the PL group, and 88.4%, 44.2%, and 44.2% in the AB group, respectively. The 6-, 12-, and 18-month PFS rates were 56.9%, 43.0%, and 32.1% in the PL group, and 74.2%, 40.9%, and 30.7% in the AB group, respectively. No significant differences were observed in both OS (HR, 0.498; 95% CI, 0.217-1.143; *P* = 0.1) and PFS (HR, 0.913; 95% CI, 0.512-0.1.629; *P* = 0.758) between the two groups. Through subgroup analysis, we developed a Cirrhosis-Portal vein invasion-ALBI (CPA) score and identified that the AB group exhibited significantly longer OS than the PL group in the CPA high population (HR, 0.219; 95% CI, 0.075–0.637; *P* = 0.005). The treatment-related adverse events between the PL group and the AB group were comparable.

**Conclusions:**

This study suggests that the efficacy of pembrolizumab-lenvatinib and atezolizumab-bevacizumab is comparable in first-line treatment of unresectable HCC, the atezolizumab-bevacizumab combination may confer additional benefits for patients with high CPA scores compared to pembrolizumab-lenvatinib.

## Introduction

Hepatocellular carcinoma (HCC) is a significant global health concern characterized by a rising incidence and mortality rate (1, 2). Despite the expanding implementation of surgical and locoregional therapies worldwide (3), it is estimated that approximately 50–60% of HCC patients ultimately require systemic treatments (4, 5). For over a decade, tyrosine kinase inhibitors (TKIs) have been the primary treatment for unresectable HCC. However, the emergence of immunotherapies, specifically immune-checkpoint inhibitors (ICIs), has revolutionized the treatments of HCC. The combination of TKIs and ICIs has become the first-line treatment for unresectable HCC, as recommended by various guidelines (6–8).

In the IMbrave-150 trial, a phase III international randomized controlled trial, patients with advanced-stage HCC receiving atezolizumab–bevacizumab demonstrated significant longer overall survival (OS) (hazard ratio [HR] = 0.66, 95% confidence interval [CI] 0.52–0.85; *P* < 0.001) and progression-free survival (PFS) (HR= 0.65, 95% CI 0.53–0.81; *P*<0.001) compared to patients receiving sorafenib (9, 10). The trial reported an objective response rate (ORR) of 35% based on the modified Response Evaluation Criteria in Solid Tumors (mRECIST), with a complete response (CR) rate of 12%. The median OS duration was 19.2 months, and the treatment exhibited a favorable safety profile. The success of the IMbrave150 trial led to the adoption of atezolizumab–bevacizumab combination as the standard-of-care first-line systemic treatment for advanced-stage HCC.

On the other hand, based on the results of the KEYNOTE-224 trial (11), an open-label, multi-centers, phase II study, pembrolizumab was approved by the American food and drug administration (FDA) for advanced HCC in patients previously treated with sorafenib. However, in the LEAP-002 trail (12), a phase III international randomized controlled trial, the improvement in OS did not reach the statistically significant difference in first-line treatment of advanced HCC. The median OS of pembrolizumab-lenvatinib vs lenvatinib was 21.1 vs 19.0 months (HR=0.84, 95% CI 0.708-0.997, *P* = 0.0227). Due to the dual endpoints of OS and PFS, the PFS test consumed part of the α efficiency, resulting in an actual p-value slightly larger than the preset p-value of 0.0185, which is close to achieving the difference in efficacy. Subgroup analysis revealed that hepatitis B virus (HBV)-related HCC patients benefited from pembrolizumab-lenvatinib treatment. As approximately 80% of HCC patients in China are infected with HBV (13), pembrolizumab-lenvatinib has been applied in the real-world first-line treatment of advanced HCC in China (14).

It is worth noting that the control group in the IMbrave-150 trial was sorafenib, whereas in the LEAP-002 trial, it was lenvatinib. According to results of the REFLECT trial (15), lenvatinib was superior to sorafenib in both OS (13.6 months vs 12.3 months, HR=0.92) and PFS (7.4 months vs 3.7 months; HR=0.66). Therefore, the relative superiority of atezolizumab–bevacizumab versus pembrolizumab-lenvatinib in treatment of advanced HCC remains unclear. The aim of this study is to compare the efficacy of atezolizumab–bevacizumab and pembrolizumab-lenvatinib as first-line treatments for HCC.

## Methods

### Patients

We retrospectively enrolled unresectable HCC patients who received pembrolizumab plus lenvatinib (PL group) or atezolizumabplus plus bevacizumab (AB group) at Sun Yat-sen University Cancer Center from January 2019 to June 2023. The diagnosis of HCC was confirmed by pathology or clinical features according to the American Association for the Study of Liver Diseases criteria (16). The inclusion criteria were as follows: (1) patients who were not suitable for curative therapies; (2) patients with Child-Pugh grade A or B; (3) patients without previous systemic therapy for HCC;(4) presence of at least one measurable lesion in liver based on mRECIST (17);(5) patients who received at least two cycles of PD1 injection and had assessable imaging surveillance data;(6) patients at 18-75 years old. The exclusion criteria were as follows: (1)a history of other malignancies; (2)a history of autoimmune disease; (3) a history of esophageal or gastric variceal bleeding; (4) incomplete clinical information. Ultimately, 72 patients were included in the PL group and 92 patients were included in the AB group.

### Treatment procedure

Patients in the PL group received lenvatinib orally in a dose of 8mg once daily (or 12mg once daily for patients with a body weight ≥60kg) and pembrolizumab intravenously in the dose of 200mg every 3 weeks (12). Patients in the AB group received atezolizumab at a dose of 1200 mg plus bevacizumab at a dose of 15 mg per kilogram of body weight intravenously every 3 weeks (10).

### Follow-up

Patients underwent surveillance at intervals of 6-8 weeks, which included enhanced CT or MRI scans and serum alpha-fetoprotein (AFP) testing to assess tumor response. The response was defined as CR, partial response (PR), stable disease (SD), or progressive disease (PD) according to the mRECIST criteria (17). The ORR represented the proportion of patients with CR and PR, and the disease control rate (DCR) defined as the sum of CR, PR, and SD.

The primary endpoints of the study OS and PFS. OS was measured from the initiation of first-line treatment until death from any cause, while PFS referred to the time between treatment initiation and disease progression. The secondary endpoints included ORR and DCR. Adverse events (AEs) were assessed according to the Common Terminology Criteria for Adverse Events (CTCAE) version 5.0.

### Statistical analysis

To minimize selection bias and potential confounders between the two groups, propensity score matching (PSM) analysis was employed. The following variables were included in the matching process: presence of extrahepatic metastases, AFP levels, and combination with interventional therapy. Categorical variables were compared using either Pearson’s chi-square test or Fisher’s exact test. Survival curves were constructed using the Kaplan-Meier method and compared using the log-rank test. Cox proportional hazard models were used to estimate HRs with a 95% CI. Variables with a significance level of P < 0.1 in the univariate analyses were included in the multivariate analyses. All statistical analyses were conducted using SPSS version 26.0 or R version 4.2.2 (R Foundation, Vienna, Austria).

## Results

### Baseline characteristics of patients

**Figure 1:**
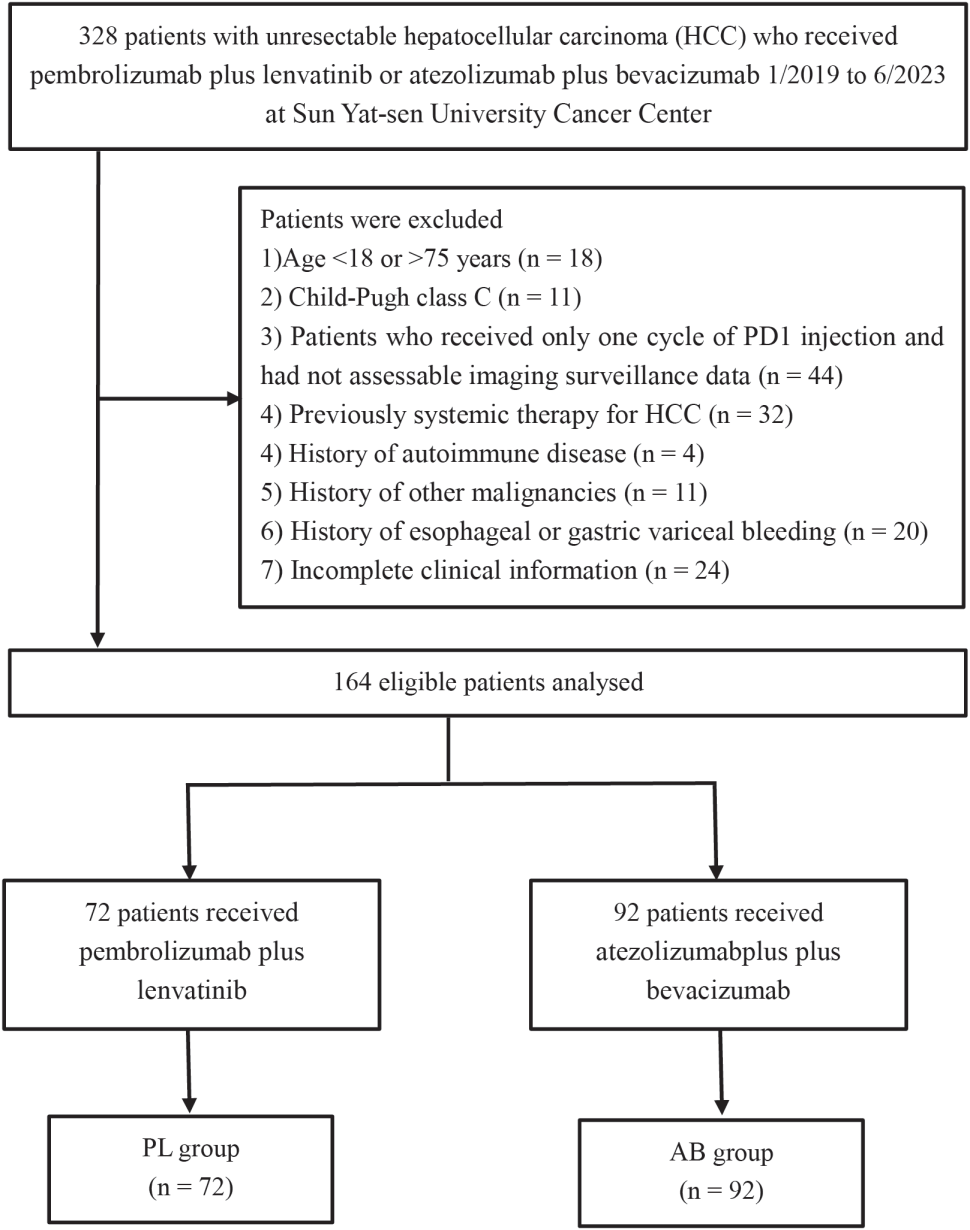
Flow diagram for the patient selection process.

In this study, a total of 72 patients receiving pembrolizumab plus lenvatinib (PL group) and 92 patients receiving atezolizumab plus bevacizumab (AB group) were included from January 2019 to June 2023 (**Figure 1**). The median duration of follow-up was 13.7 months (95% CI, 12.0-15.5). Compared to the PL group, the AB group showed more patients without extrahepatic metastases (64.1% vs 44.4%) and with combination of interventional therapy (88.0% vs 55.6%). After PSM, there were no significant differences in the baseline characteristics between the two groups (**Table 1**).

**Table 1:** Baseline characteristics of patients in entire cohort and PSM cohort.

	Entire cohort			PSM cohort		
	PL group	AB group	P-value	PL group	AB group	P-value
	(n=72)	(n=92)		(n=51)	(n=51)	
Age, years			0.651			0.543
<60	42 (58.3%)	58 (63.0%)		33 (64.7%)	29 (56.9%)	
≥60	30 (41.7%)	34 (37.0%)		18 (35.3%)	22 (43.1%)	
Gender			0.729			1.000
male	62 (86.1%)	82 (89.1%)		46 (90.2%)	45 (88.2%)	
female	10 (13.9%)	10 (10.9%)		5 (9.8%)	6 (11.8%)	
HBV infection			0.184			0.327
absence	15 (20.8%)	11 (12.0%)		13 (25.5%)	8 (15.7%)	
presence	57 (79.2%)	81 (88%)		38 (74.5%)	43 (84.3%)	
Cirrhosis			0.667			0.841
absence	47 (65.3%)	56 (60.9%)		31 (60.8%)	29 (56.9%)	
presence	25 (34.7%)	36 (39.1%)		20 (39.2%)	22 (43.1%)	
Tumor diameter, cm			0.454			0.843
<10	38 (52.8%)	42 (45.7%)		25 (49.0%)	27 (52.9%)	
≥10	34 (47.2%)	50 (54.3%)		26 (51.0%)	24 (47.1%)	
Tumor number			1.000			0.820
single	18 (25.0%)	24 (26.1%)		14 (27.5%)	12 (23.5%)	
mutiple	54 (75.0%)	68 (73.9%)		37 (72.5%)	39 (76.5%)	
Portal vein invasion			0.920			0.211
no	32 (44.4%)	28 (30.4%)		21 (41.2%)	14 (27.5%)	
yes	40 (55.6%)	64 (69.6%)		30 (58.8%)	37 (72.5%)	
Extrahepatic metastases			0.018			1.000
no	32 (44.4%)	59 (64.1%)		29 (56.9%)	29 (56.9%)	
yes	40 (55.6%)	33 (35.9%)		22 (43.1%)	22 (43.1%)	
BCLC stage			0.785			0.288
B	12 (16.7%)	18 (19.6%)		11 (21.6%)	6 (11.8%)	
C	60 (83.3%)	74 (80.4%)		40 (78.4%)	45 (88.2%)	
Platelet, x10^9^/L			0.889			0.695
>100	66 (91.7%)	86 (93.5%)		48 (94.1%)	47 (92.2%)	
≤100	6 (8.3%)	6 (6.5%)		3 (5.9%)	4 (7.8%)	
ALT, U/L			0.905			0.843
< 40	36 (50.0%)	44 (47.8%)		27 (52.9%)	25 (49.0%)	
≥ 40	36 (50.0%)	48 (52.2%)		24 (47.1%)	26 (51.0%)	
AST, U/L			1.000			0.645
< 40	21 (29.2%)	27 (29.3%)		11 (21.6%)	14 (27.5%)	
≥ 40	51 (70.8%)	65 (70.7%)		40 (78.4%)	37 (72.5%)	
AFP, ng/mL			0.056			1.000
<400	43 (59.7%)	40 (43.5%)		27 (52.9%)	27 (52.9%)	
≥400	29 (40.3%)	52 (56.5%)		24 (47.1%)	24 (47.1%)	
TBIL, μmol/L			0.265			0.410
< 20.5	50 (69.4%)	55 (59.8%)		35 (68.6%)	30 (58.8%)	
≥20.5	22 (30.6%)	37 (40.2%)		16 (31.4%)	21 (41.2%)	
ALB, g/L			0.839			0.388
>35	65 (90.3%)	81 (88.0%)		46 (90.2%)	42 (82.4%)	
≤35	7 (9.7%)	11 (12.0%)		5 (9.8%)	9 (17.6%)	
ALBI			0.238			0.675
grade 1	28 (38.9%)	26 (28.3%)		18 (35.3%)	14 (27.5%)	
grade 2	41 (56.9%)	58 (63.0%)		30 (58.8%)	33 (64.7%)	
grade 3	3 (4.2%)	8 (8.7%)		3 (5.9%)	4 (7.8%)	
Combination with interventional therapy			<0.001			1.000
no	32 (44.4%)	11 (12.0%)		11 (21.6%)	11 (21.6%)	
yes	40 (55.6%)	81 (88.0%)		40 (78.4%)	40 (78.4%)	

Categorical variables are described as frequencies and percentages. AFP, alpha fetoprotein; ALB, albumin; ALBI, Albumin-Bilirubin; ALT, alanine aminotransferase; AST, aspartate aminotransferase; HBV, hepatitis B virus; PT, prothrombin time; TBIL, total bilirubin.

### Overall survival analysis between the PL and AB groups

**Figure 2:**
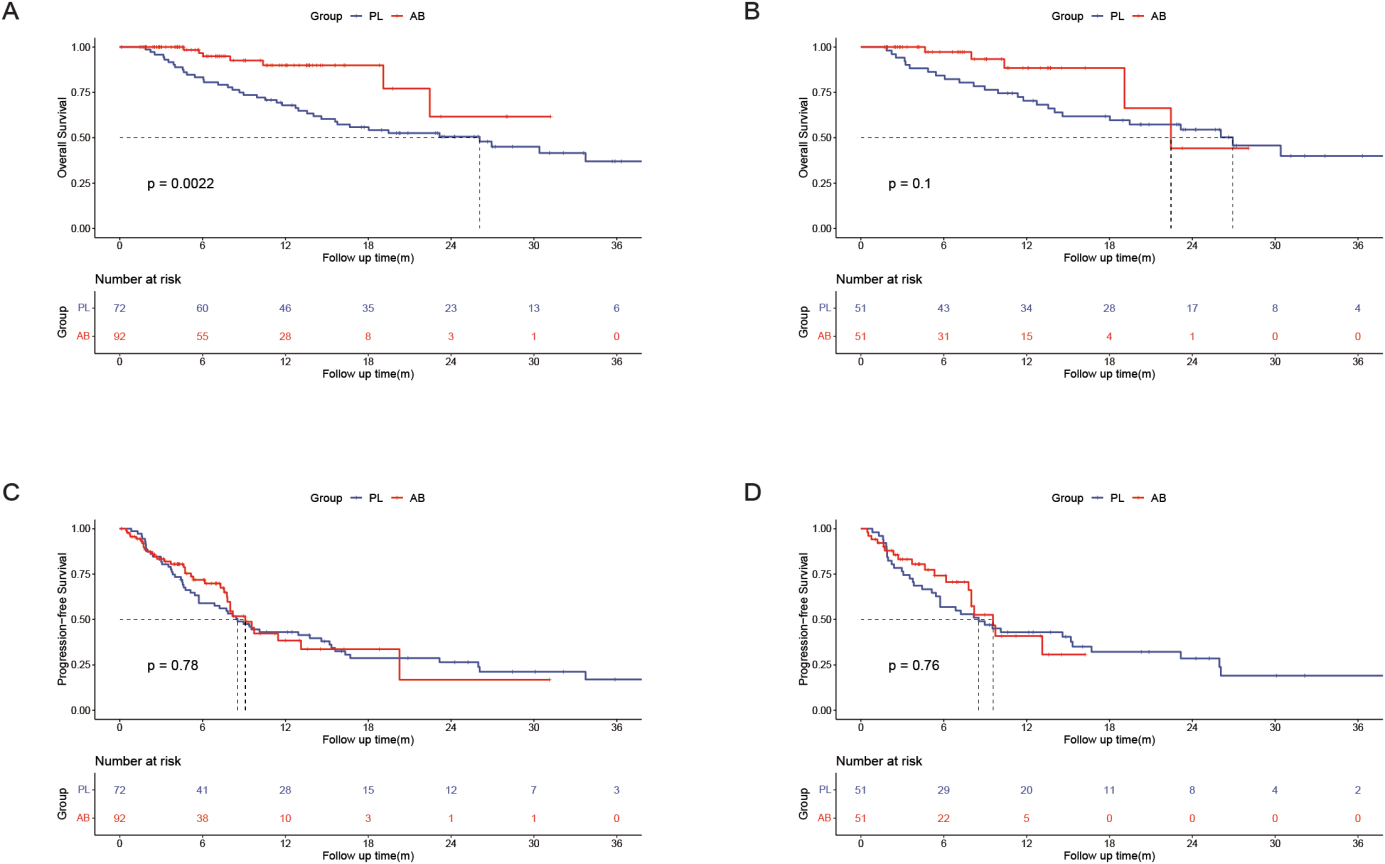
Kaplan–Meier curves of overall survival between the PL group and the AB group in the entire cohort **(A)** and the PSM cohort**(B)**; Kaplan–Meier curves of progression-free survival between the PL group and the AB group in the entire cohort **(C)** and the PSM cohort **(D)**.

In the entire cohort, the median OS was 26.1(95% CI, 14.0-38.1) months in the PL group and unreached in the AB group. The 1-, 2-, and 3-year OS rates were 67.9%, 50.6%, and 37.0% in the PL group, and 89.9%, 61.7%, and 61.7% in the AB group. In the PSM cohort, the median OS was 26.9 (95% CI, 16.6-37.3) months in the PL group and 22.5 (95% CI, 15.8-29.1) months in the AB group. The 1-, 2-, and 3-year OS rates were 70.4%, 54.5%, and 40.0% in the PL group, and 88.4%, 44.2%, and 44.2% in the AB group. The AB group exhibited a significantly longer OS than the PL group in the entire cohort (HR, 0.368; 95% CI, 0.194-0.698; *P* = 0.002; **Figure 2A**) but there was no significant difference between the two groups in the PSM cohort (HR, 0.498; 95% CI, 0.217-1.143; *P* = 0.1; **Figure 2B**).

In the entire cohort, 1(1.4%) patient had CR, 23(31.9%) patients had PR, 33(45.8%) patients had SD, 15(20.8%) patients had PD, and 2(2.8%) patients received surgical resection due to effective treatment in the PL group; 4(4.3%) patients had CR, 29(31.5%) patients had PR, 47(51.1%) patients had SD, 12(13.0%) patients had PD, and 5(5.4%) patients received surgical resection due to effective treatment in the PL group; there was no significant difference in ORR between the PL group and the AB group (33.3% vs 35.9%, *P* = 0.862). In the PSM cohort, 18(35.3%) patients had PR, 21(41.2%) patients had SD, 12(23.5%) patients had PD, and 2(3.9%) patients received surgical resection due to effective treatment in the PL group; 18(35.3%) patients had PR, 26(51.0%) patients had SD, 7(13.7%) patients had PD, and 1(2.0%) patient received surgical resection due to effective treatment in the PL group; there was no significant difference in ORR between the PL group and the AB group (35.3% vs 35.3%, *P* = 1.000). Details were described in the **Table 2**.

**Table 2:** The best response between the PL group and the AB group.

Evaluation (mRECIST)	Entire cohort			PSM cohort		
	PL group (n=72)	AB group (n=92)	P-value	PL group (n=51)	AB group (n=51)	P-value
Complete response	1 (1.4%)	4 (4.3%)	——	0 (0%)	0 (0%)	——
Partial response	23 (31.9%)	29 (31.5%)	——	18 (35.3%)	18 (35.3%)	——
Stable disease	33 (45.8%)	47 (51.1%)	——	21 (41.2%)	26 (51.0%)	——
Progressive disease	15 (20.8%)	12 (13.0%)	——	12 (23.5%)	7 (13.7%)	——
Objective response rate	24 (33.3%)	33 (35.9%)	0.862	18 (35.3%)	18 (35.3%)	1.000
Disease control rate	57 (79.2%)	80 (87.0%)	0.262	39 (76.5%)	44 (86.3%)	0.309
Surgical resection	2 (2.8%)	5 (5.4%)	0.443	2 (3.9%)	1 (2.0%)	0.558

mRECIST, modified response evaluation criteria in solid tumors.

Univariate analyses identified extrahepatic metastases (HR, 1.876; 95% CI, 1.036-3.396; *P* = 0.38) and treatment with atezolizumab plus bevacizumab (HR, 0.295; 95% CI, 0.129-0.673; *P* = 0.004) as the independent risk factors associated with OS. Factors with a p-value less than 0.1 in the univariate analyses were included in the multivariate analyses. The multivariate analyses identify age (HR, 1.653; 95% CI, 0.920-2.968; *P* = 0.093), extrahepatic metastases (HR, 1.668; 95% CI, 0.917-3.034; *P* = 0.094), and treatment with atezolizumab plus bevacizumab (HR, 0.327; 95% CI, 0.143-0.751; *P* = 0.008) as the independent risk factors associated with OS. More details were described in the **Table 3**.

**Table 3:** Univariate and multivariate analysis of risk factors for overall survival and recurrence-free survival.

Variables	Overall survival						Recurrence-free survival					
	Univariate analysis			Multivariate analysis			Univariate analysis			Multivariate analysis		
	HR	95% CI	*P*	HR	95% CI	*P* value	HR	95% CI	*P*	HR	95% CI	*P*
Age, years (<60: ≥60)	1.663	0.927-2.986	0.088	1.653	0.920-2.968	0.093	1.177	0.769-1.801	0.453			
Gender (female: male)	1.112	0.495-2.496	0.798				0.925	0.491-1.743	0.808			
Tumor diameter, cm (<10 vs ≥10)	1.048	0.583-1.884	0.876				1.158	0.760-1.764	0.495			
Tumor number (solitary: multiple)	1.677	0.826-3.405	0.152				2.070	1.212-3.535	0.008	2.171	1.268-3.717	0.005
Cirrhosis (absence vs presence)	0.584	0.301-1.132	0.111				0.652	0.414-1.029	0.066			
Extrahepatic metastases (no vs yes)	1.876	1.036-3.396	0.038	1.668	0.917-3.034	0.094	1.275	0.836-1.946	0.259			
Portal vein invasion (no vs yes)	0.872	0.483-1.575	0.650				0.761	0.498-1.161	0.205			
HBV infection (no: yes)	0.990	0.461-2.129	0.980				0.764	0.443-1.320	0.335			
Platelet, ×109/L (>100 vs ≤100) (≥100:<100)	0.438	0.106-1.814	0.255				0.848	0.369-1.946	0.697			
AFP, ng/mL (<400 vs ≥400)	0.846	0.465-1.539	0.583				1.132	0.740-1.733	0.567			
ALT, U/L (< 40 vs ≥ 40)	1.322	0.734-2.380	0.353				0.883	0.578-1.348	0.564			
AST, U/L (< 40 vs ≥ 40)	1.350	0.701-2.602	0.370				1.128	0.709-1.793	0.611			
ALB, g/L (>35 vs ≤35)	1.548	0.608-3.942	0.360				1.138	0.549-2.360	0.727			
TBIL, μmol/L (≤20.5:>20.5)	1.207	0.647-2.252	0.554				1.154	0.743-1.791	0.523			
ALBI grade (1: 2,3)	1.143	0.685-1.906	0.608				1.133	0.786-1.632	0.503			
BCLC stage (B: C)	1.224	0.586-2.555	0.590				0.826	0.504-1.352	0.446			
Interventional therapy (no: yes)	0.611	0.334-1.115	0.108				0.990	0.625-1.568	0.967			
Group (AB vs PL)	0.295	0.129-0.673	0.004	0.327	0.143-0.751	0.008	0.939	0.601-1.465	0.781			

HR, hazard rate; CI, confidence interval; HBV, hepatitis B virus; AFP, alpha fetoprotein; ALT, alanine aminotransferase; AST, aspartate aminotransferase; PT, prothrombin time; ALB, albumin; TBIL, total bilirubin; ALBI, Albumin-Bilirubin; PVTT, portal vein tumor thrombus; HAIC, hepatic arterial infusion chemotherapy.

### Progression-free survival between the PL and AB groups

In the entire cohort, the median PFS was 8.5(95% CI, 6.2-10.9) months in the PL group and 9.1(95% CI, 7.5-10.7) months in the AB group. The 6-, 12-, and 18-month PFS rates were 59.0%, 43.0%, and 28.7% in the PL group, and 71.8%, 38.4%, and 16.8% in the AB group. In the PSM cohort, the median PFS was 8.5(95% CI, 4.2-12.9) months in the PL group and 9.6(95% CI, 7.5-11.7) months in the AB group. The 6-, 12-, and 18-month PFS rates were 56.9%, 43.0%, and 32.1% in the PL group, and 74.2%, 40.9%, and 30.7% in the AB group. There was no significant difference in PFS between the two groups in the entire cohort (HR, 0.939; 95% CI, 0.602-0.1.464; *P* = 0.781; **Figure 2C**) and PSM cohort (HR, 0.913; 95% CI, 0.512-0.1.629; *P* = 0.758; **Figure 2D**).

Univariate analyses identified tumor number (HR, 2.070; 95% CI, 1.212-3.535; *P* = 0.008) as the independent risk factors associated with PFS. Factors with a p-value less than 0.1 in the univariate analyses were included in the multivariate analyses. The multivariate analyses identified tumor number (HR, 2.171; 95% CI, 1.268-3.717; *P* = 0.005) as the independent risk factors associated with PFS. Details were described in the **Table 3**.

### Subgroup analysis

**Figure 3:**
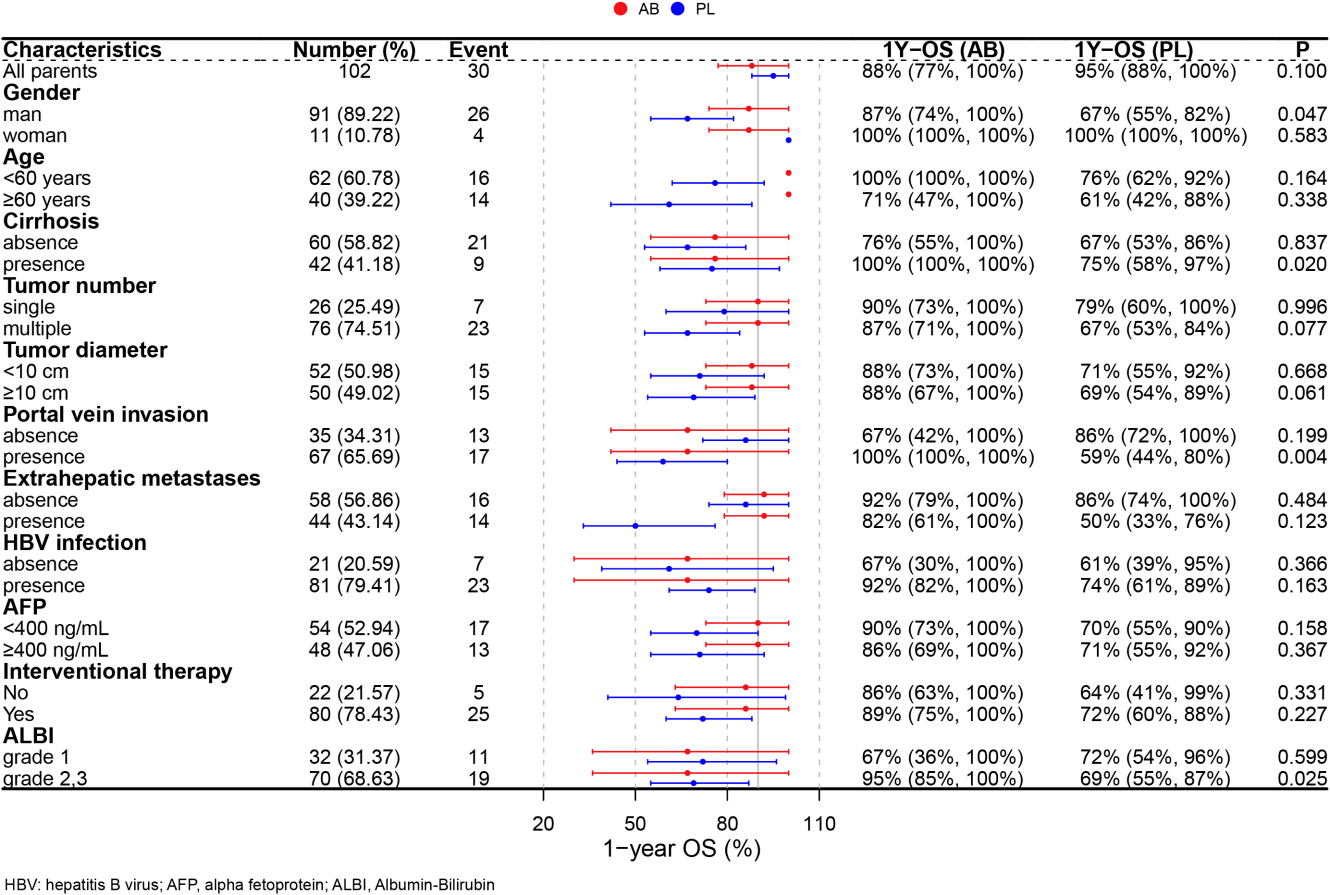
Forest plots for subgroup analysis.

**Figure 4:**
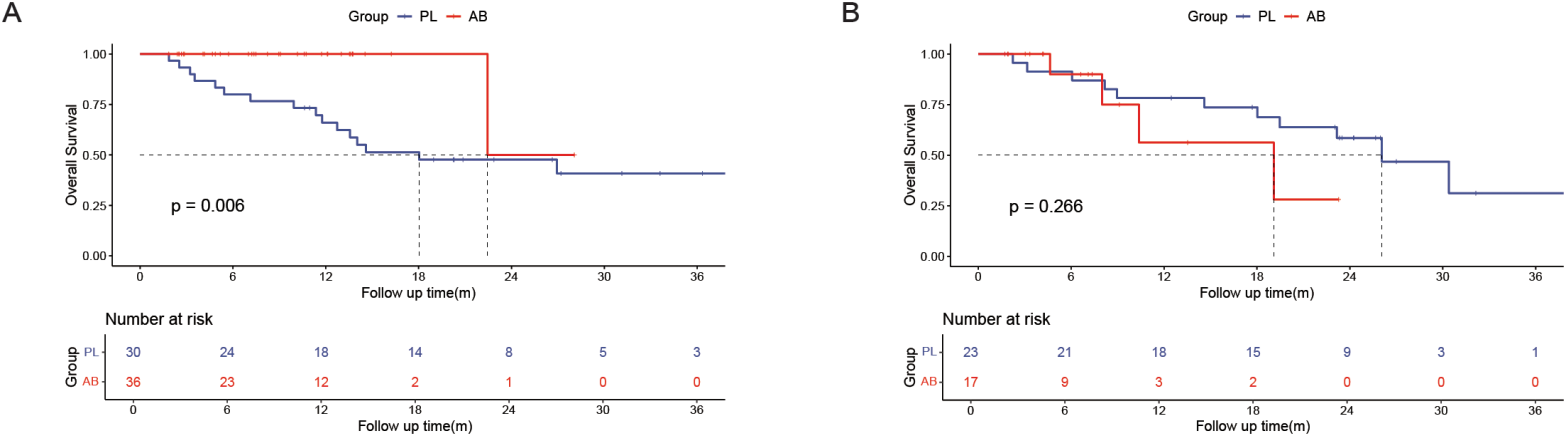
Kaplan–Meier curves of overall survival between the PL group and the AB group in the CPA high population **(A)** and CPA low population **(B)**.

A subgroup analysis was performed in the PSM cohort to further explore the effects of treatment (**Figure 3**). The analysis revealed that the use of atezolizumab plus bevacizumab was associated with improved survival in following subgroups: male (*P* = 0.047), presence of cirrhosis (*P* = 0.020), presence of portal vein invasion (*P* = 0.004), and ALBI grade 2/3 (*P* = 0.025). To provide a more accurate characterization of the population that benefits from this treatment, we established a Cirrhosis-Portal vein invasion-ALBI (CPA) score based on the results of subgroup analysis: absence of cirrhosis, or absence of portal vein invasion were calculated as 0 score; presence of cirrhosis, or presence of portal vein invasion were calculated as 1 score; ALBI grade 1,2,3 were calculated as 1,2,3 score. Patients with scores 1 and 2 were classified as the CPA low population, while those with scores ranging from 3 to 5 were categorized as the CPA high population (**Table 4**). Analysis revealed a significant improvement in OS for the AB group compared to the PL group in the CPA high population (HR, 0.232; 95% CI, 0.082–0.655; *P* = 0.006; **Figure 4A**). However, no significant difference in overall survival was observed between the two groups in the CPA low population (HR, 2.28; 95% CI, 0.533–9.73; *P* = 0.266; **Figure 4B**).

**Table 4:** Cirrhosis-Portal vein invasion-ALBI (CPA) score in entire cohort and PSM cohort.

	Entire cohort		PSM cohort	
CAP score	PL group	AB group	PL group	AB group
	(n=72)	(n=92)	(n=51)	(n=51)
1	13 (18.1%)	10 (10.9%)	8 (15.7%)	3 (5.9%)
2	22 (30.1%)	19 (20.7%)	13 (25.5%)	12 (23.5%)
3	24 (33.3%)	37 (40.2%)	20 (39.2%)	21 (41.2%)
4	10 (13.9%)	23 (25.0%)	7 (13.7%)	14 (27.5%)
5	3 (4.2%)	3 (3.7%)	3 (5.9%)	1 (2.0%)

### Safety

Overall, there were no significant differences in treatment-related AEs between the PL group and the AB group (**Table 5**). The most common somatosensory AEs were decreased appetite (19.6% vs 7.8%, *P =* 0.150) and pain (13.7% vs 7.8%, *P =* 0.523) in both PL group and AB group. The most frequently observed laboratory-related AEs were increased total bilirubin (33.3% vs. 23.5%, *P* = 0.380) and increased aspartate aminotransferase (23.5% vs. 25.5%, *P* = 1.000) in both the PL and AB groups. The incidence of grade 3 and 4 AEs was comparable between the two groups. No AEs-associated death was observed during the follow-up period. AEs were manageable because of effective supportive care, including analgesic therapy, hepatic functional protection, and so on.

**Table 5:** Treatment-related adverse events between the PL group and the AB group.

Treatment-related adverse events, n (%)	Any grade			Grade 3/4		
	PL Group (n=51)	AB Group (n=51)	*P*-value	PL Group (n=51)	AB Group (n=51)	*P*-value
Rash	2 (3.9%)	1 (2.0%)	0.558	0 (0%)	0 (0%)	NA
Pruritus	2 (3.9%)	0 (0%)	0.153	0 (0%)	0 (0%)	NA
Pain	7 (13.7%)	4 (7.8%)	0.523	0 (0%)	0 (0%)	NA
Fever	3 (5.9%)	1 (2.0%)	0.308	0 (0%)	0 (0%)	NA
Diarrhea	4 (7.8%)	1 (2.0%)	0.169	0 (0%)	0 (0%)	NA
Fatigue	1 (2.0%)	3 (5.9%)	0.308	0 (0%)	0 (0%)	NA
Nausea	2 (3.9%)	0 (0%)	0.153	0 (0%)	0 (0%)	NA
Decreased appetite	10 (19.6%)	4 (7.8%)	0.150	0 (0%)	0 (0%)	NA
Cough	3 (5.9%)	2 (3.9%)	0.647	0 (0%)	0 (0%)	NA
Alimentary tract hemorrhage	0 (0%)	0 (0%)	NA	0 (0%)	0 (0%)	NA
Edema peripheral	1 (2.0%)	3 (5.9%)	0.308	0 (0%)	0 (0%)	NA
Hypothyroidism	3 (5.9%)	3 (5.9%)	1.000	0 (0%)	0 (0%)	NA
Hyperthyroidism	0 (0%)	0 (0%)	NA	0 (0%)	0 (0%)	NA
Hypertension	0 (0%)	0 (0%)	NA	0 (0%)	0 (0%)	NA
Laboratory-related AEs, n (%)						
Hemoglobin decreased	4 (7.8%)	3 (5.9%)	0.695	1 (2.0%)	0 (0%)	0.315
Platelet count decreased	6 (11.8%)	7 (13.7%)	1.000	1 (2.0%)	1 (2.0%)	1.000
Alanine aminotransferase increased	2 (3.9%)	5 (9.8%)	0.240	1 (2.0%)	3 (5.9%)	0.308
Aspertate aminotransferase increased	12 (23.5%)	13 (25.5%)	1.000	4 (7.8%)	8 (15.7%)	0.357
Total bilirubin increased	17 (33.3%)	12 (23.5%)	0.380	6 (11.8%)	2 (3.9%)	0.141
Albumin decreased	3 (5.9%)	6 (11.8%)	0.295	0 (0%)	0 (0%)	NA
Creatinine increased	1 (2.0%)	1 (2.0%)	1.000	0 (0%)	0 (0%)	NA

## Discussion

The present study compared the efficacy and safety of pembrolizumab-lenvatinib and atezolizumab-bevacizumab in the first-line treatment of unresectable HCC. We analyzed a cohort of 72 patients receiving pembrolizumab-lenvatinib and 92 patients receiving atezolizumab-bevacizumab. We found that the AB group showed significantly longer OS than the PL group, but there was no significant difference in PFS between the two groups in the entire cohort. After PSM, there was no significant difference in both OS and PFS between the two groups. The ORR and DCR were also similar between the two groups, both in the entire cohort and PSM cohort (**Table 2**). We further performed subgroup analysis in the PSM cohort, the analysis revealed that the use of atezolizumab plus bevacizumab was associated with improved survival in following subgroups: presence of cirrhosis, presence of portal vein invasion, and ALBI grade 2/3. Based on these results, we established a Cirrhosis-Portal vein invasion-ALBI (CPA) score, which aimed to accurately identify the population that would benefit from atezolizumab-bevacizumab. We found that the AB group significantly prolonged OS compared to the PL group in the CPA high-risk population, while there was no significant difference in OS between the two groups in the CPA low-risk population. For safety, there was no significant difference in treatment-related AEs between the PL group and the AB group.

In the REFLECT trail, the median OS of patients with lenvatinib was 13.6 months, but it was 19.0 months in the LEAP-002 trail, which was much longer than expected. This might be one of reasons causing the negative results. Although the negative results of LEAP-002, the median OS of patients receiving pembrolizumab-lenvatinib was 21.2 months, even longer than the median OS (19.2 months) of patients receiving atezolizumab-bevacizumab in the IMbrave-150 trail, and the baseline characteristics of the global population enrolled in the LEAP-002 trail were similar to those in the IMbrave150 trial. Furthermore, subgroup analysis indicated that pembrolizumab-lenvatinib benefited HBV-related HCC. In this study, 88% patients in the AB group were infected with HBV. These might explain why the OS of patients with pembrolizumab-lenvatinib was similar to atezolizumab-bevacizumab in present study. Of course, the selection bias because of small samples in this study also might also be one of the reasons, although we performed PSM to reduce the influence of cofounder factors.

We further performed subgroup analysis and found that patients with male, cirrhosis, portal vein invasion, and ALBI grade 2/3 showed better OS in the AB group compared to the PL group. To accurately identify the population that would benefit from atezolizumab-bevacizumab, we established the CPA score based on the results of subgroup analysis. As we all know, cirrhosis and ALBI grade 2/3 indicate worse liver functional reserve, which are associated with poor OS in patients with HCC (18–21). Interestingly, we observed a lower incidence of bilirubin increase in the AB group compared to the PL group (any grade: 23.5% vs. 33.3%; grade 3/4: 3.9% vs. 11.8%), although the differences were not statistically significant. This suggests that atezolizumab-bevacizumab may have less impact on liver function, making it more beneficial for patients with worse liver functional reserve. Besides liver functional reserve, the CPA score incorporates portal vein invasion, which are known high-risk factors for HCC (22). The three factors included in the CPA score are routinely available data for clinicians, and this score represents the first clinical tool that helps identify the population that would benefit from atezolizumab-bevacizumab, enabling clinicians to make more suitable therapeutic regimens for their patients.

In general, there was no significant difference in treatment-related AEs between the PL group and the AB group (**Table 4**). Less patients suffered from decreased appetite (7.8% vs 19.6%) and total bilirubin increased (23.5% vs 33.3%) in the AB group than in the PL group, although the P-values were lager than 0.05. And we found more patients suffered from aspartate aminotransferase (AST) increased and total bilirubin increased in present study than previous studies, it was because a part of patients in present study received interventional therapy meanwhile.

However, it is important to acknowledge several limitations of our study. Firstly, this study is a retrospective study with a small sample size, which inevitably introduces selection bias, even though we performed PSM to reduce the influence of confounding factors. Secondly, the inclusion of patients receiving combined interventional therapy in our study raises the question of whether the conclusions can be applied to patients who only receive atezolizumab-bevacizumab or pembrolizumab-lenvatinib, which requires further exploration. Lastly, because the study was retrospective in nature, the documentation of treatment-related AEs was not comprehensive, resulting in potential inaccuracies in the data, particularly regarding somatosensory AEs.

In conclusion, our study demonstrates that pembrolizumab-lenvatinib and atezolizumab-bevacizumab exhibit comparable efficacy in the first-line treatment of unresectable HCC. However, the CPA high-risk population may benefit more from atezolizumab-bevacizumab compared to pembrolizumab-lenvatinib. The CPA score, which incorporates cirrhosis, portal vein invasion, and ALBI grade, can help clinicians identify the population that would benefit most from atezolizumab-bevacizumab. Further studies with larger sample sizes and prospective designs are warranted to validate these findings and optimize treatment strategies for HCC patients.

## Data Availability

The raw data supporting the conclusions of this article will be made available by the authors, without undue reservation.
